# Inhibition of protein kinase C increases *Prdm14* level to promote self-renewal of embryonic stem cells through reducing Suv39h-induced H3K9 methylation

**DOI:** 10.1016/j.jbc.2024.105714

**Published:** 2024-02-02

**Authors:** Junxiang Ji, Jianjian Cao, Peng Chen, Ru Huang, Shou-Dong Ye

**Affiliations:** Center for Stem Cell and Translational Medicine, School of Life Sciences, Anhui University, Hefei, Anhui, PR China

**Keywords:** self-renewal, PKC, Suv39h1, Prdm14, Dnmt3

## Abstract

Inhibition of protein kinase C (PKC) efficiently promoted the self-renewal of embryonic stem cells (ESCs). However, information about the function of PKC inhibition remains lacking. Here, RNA-sequencing showed that the addition of Go6983 significantly inhibited the expression of *de novo* methyltransferases (*Dnmt3a* and *Dnmt3b*) and their regulator *Dnmt3l*, resulting in global hypomethylation of DNA in mouse ESCs. Mechanistically, PR domain-containing 14 (*Prdm14*), a site-specific transcriptional activator, partially contributed to Go6983-mediated repression of *Dnmt3* genes. Administration of Go6983 increased *Prdm14* expression mainly through the inhibition of PKCδ. High constitutive expression of *Prdm14* phenocopied the ability of Go6983 to maintain` mouse ESC stemness in the absence of self-renewal-promoting cytokines. In contrast, the knockdown of *Prdm14* eliminated the response to PKC inhibition and substantially impaired the Go6983-induced resistance of mouse ESCs to differentiation. Furthermore, liquid chromatography–mass spectrometry profiling and Western blotting revealed low levels of Suv39h1 and Suv39h2 in Go6983-treated mouse ESCs. Suv39h enzymes are histone methyltransferases that recognize dimethylated and trimethylated histone H3K9 specifically and usually function as transcriptional repressors. Consistently, the inhibition of *Suv39h1* by RNA interference or the addition of the selective inhibitor chaetocin increased *Prdm14* expression. Moreover, chromatin immunoprecipitation assay showed that Go6983 treatment led to decreased enrichment of dimethylation and trimethylation of H3K9 at the *Prdm14* promoter but increased RNA polymerase Ⅱ binding affinity. Together, our results provide novel insights into the pivotal association between PKC inhibition-mediated self-renewal and epigenetic changes, which will help us better understand the regulatory network of stem cell pluripotency.

Embryonic stem cells (ESCs), isolated from the inner cell mass of the developing blastocyst, proliferate indefinitely in the undifferentiated state and retain the capacity to differentiate into somatic cells when they receive the appropriate signals (1). Murine ESCs (mESCs) were established initially in 1981 and maintained on murine embryonic fibroblast layer feeders (2, 3); these cells can be replaced by leukemia inhibitory factor (LIF) in serum-containing medium (4, 5). LIF stimulates Stat3 to promote the self-renewal of mESCs, and many downstream targets have been identified; however, only the knockdown of *Tfcp2l1* impaired the function of the LIF/Stat3 signaling pathway (6, 7, 8). In 2008, Ying *et al*. reported that the application of two specific inhibitors of glycogen synthase kinase 3 and mitogen-activated protein kinase kinase, CHIR99021 and PD0325901 (also known as 2I), enables robust replication of mESCs in serum-free conditions (9). *Esrrb* is the key target of CHIR99021, while PD0325901 functions mainly by stabilizing the Klf2 protein (10, 11). 2I has been successfully used to establish rat ESCs *in vitro* (12, 13). However, neither LIF nor 2I is suitable for the establishment and maintenance of human ESCs, which were eventually derived in 1998 and require cytokines activin A and basic FGF (bFGF) when grown on murine embryonic fibroblasts or specific substrates such as Matrigel or laminin (14). Nanog has been shown to be an important candidate for activin A and bFGF (15, 16). However, the culture conditions mentioned above are not sufficient to sustain the pluripotency of ESCs from other animals. Identifying new conditions and furthering the understanding of the associated mechanisms have become popular and difficult points in the field of regenerative medicine.

To address this issue, much work has been done to explore the optimal factors and environment for culturing ESCs *in vitro*, and much progress has been made, especially in mESCs, for which Go6983 and GF109203X are both selective inhibitors of protein kinase C (PKC) and have the ability to promote ESC maintenance (17, 18, 19). PKCs belong to the family of serine/threonine kinases and are composed of at least 10 ubiquitously expressed isoenzymes subdivided into three subgroups according to their activation requirements (1): classical PKCs, PKC α, β I, β II, and γ require calcium, phosphatidylserine, and diacylglycerol for their activation (2); novel PKCs, δ, ε, η, and θ are calcium-independent and require the same lipids as classical PKCs for their activation; and (3) atypical PKCs, ζ and λ/ι are both calcium-independent and DAG-insensitive and can be activated by different lipids, such as ceramide (20). PKC family members regulate a large number of physiological processes, including cell growth and differentiation (21). Activation of PKC induces epithelial-mesenchymal transition, a marker of early differentiation, and promotes cardiogenesis in ESCs (22, 23). However, the inhibition of pan-PKCs with selective inhibitors efficiently promoted the derivation and self-renewal of ESCs isolated from multiple species, such as mice, rats, and humans (17, 18, 19). Among the PKC isoforms, PKCζ is crucial for inducing multilineage differentiation in mouse and rat ESCs (17, 18, 24). A few substrates of PKC β II and Ⅰ have been identified in mESCs, such as hnRNP C1/C2, nucleophosmin 1, SRP20, β-actin, hnRNPK, RbAp48, SAE1, eIF-3, Baf53a, HIF-1α, NUMB, and Notch1 (25, 26, 27). The changes in the modification and intracellular localization of these proteins may be associated with the self-renewal of ESCs mediated by PKC inhibition. However, there is little information on the specific roles of these genes in undifferentiated ESCs in the presence of PKC inhibitors. In addition, whether there are other downstream mechanisms that can mediate the maintenance of ESCs regulated by the repression of PKC, especially at the transcriptional level, are not well understood.

Here, through RNA sequencing and dot blot analysis, we found that the PKC inhibitor Go6983 suppressed the expression of *Dnmt3* family genes, thereby maintaining the hypomethylation state of mESCs. Further studies revealed that Go6983 induced the expression of the *Prdm14* gene through the inhibition of Suv39h enzymes-mediated dimethylation and trimethylation of H3K9. Knockdown of *Prdm14* partially impaired the Go6983-mediated transcription of *Dnmt3* family genes and self-renewal of ESCs. These findings expand our understanding of the regulatory network involved in stem cell pluripotency.

## Results

### Go6983 promotes short-term self-renewal of mESCs in serum-free conditions

**Figure 1:**
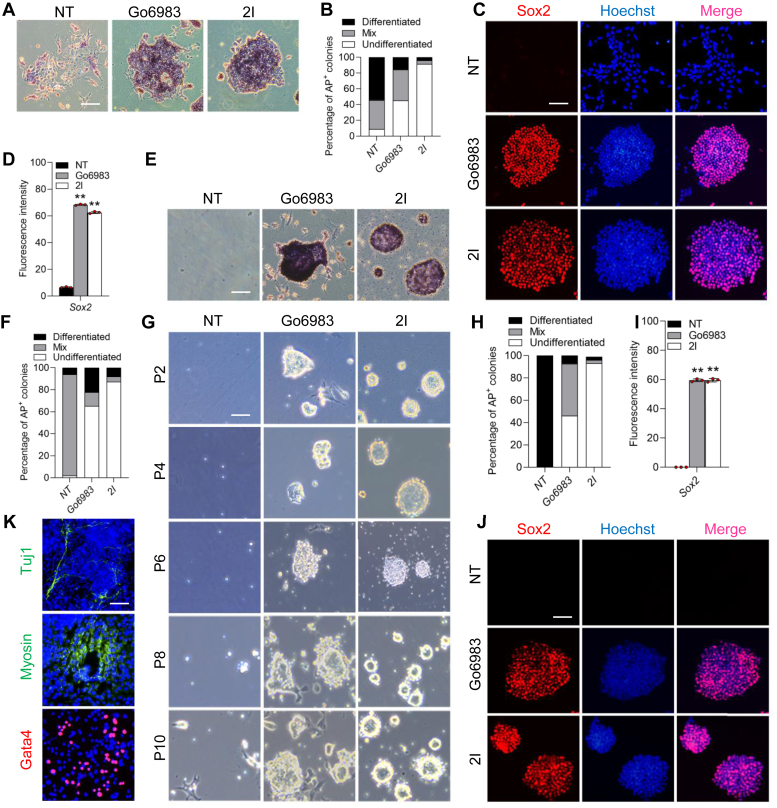
**Go6983 promotes short-term self-renewal of mESCs in serum-free conditions**. *A*, AP staining of 46C mESCs cultured in serum-containing medium supplemented with 5 μM Go6983 or 2I (3 μM CHIR99021 and 1 μM PD0325901) for 8 days. Scale bar, 100 μm. *B*, quantification of AP-positive colonies in (*A*). *C*, immunofluorescence analysis of Sox2 in mESCs treated with Go6983 or 2I for 8 days in serum-containing medium. Scale bar, 100 μm. *D*, quantification of fluorescence intensity (*red*) in (*C*). The data are presented as the mean ± SD (N = 3 biological replicates). ∗∗*p* < 0.01 *versus* NT, as determined by one-way ANOVA with Sidak’s multiple comparisons test. *E*, AP staining of 46C mESCs cultured in serum-free medium in the presence of Go6983 or 2I after one generation. Scale bar, 100 μm. *F*, quantification of AP-positiIcolonies in (*E*). *G*, morphology of 46C mESCs cultured in N2B27 supplemented with Go6983 or 2I for different passages. Scale bar, 100 μm. P, passage*. H*, the colonies in (*G*) were stained by using AP staining kit and then quantified. *I*, quantification of fluorescence intensity (*red*) in (*J*). The data are presented as the mean ± SD (N = 3 biological replicates). ∗∗*p* < 0.01 *versus* NT, as determined by one-way ANOVA with Sidak’s multiple comparisons test. *J*, immunofluorescence staining for Sox2 in cells treated with Go6983 or 2I cultured in N2B27 for 10 passages. Scale bar, 100 μm. *K*, immunofluorescence staining of Gata4, myosin, and Tuj1 in EB-derived cells. Scale bar, 100 μm. AP, alkaline phosphatase; EB, embryoid body; mESCs, mouse embryonic stem cells; NT, no treatment.

Like in Section 2I, the addition of Go6983, a selective inhibitor of pan-PKC, promoted mESC self-renewal in the absence of LIF in the serum-containing medium; these cells could be continually passaged, generated additional alkaline phosphatase (AP) colonies, and expressed high levels of Sox2, a pluripotency marker (Fig. 1, *A*–*D*), whereas, no treatment (NT) control cells differentiated (Fig. 1, *A*–*D*), indicating that inhibition of the pan-PKC can recapitulate the ability of LIF to support mESC self-renewal under serum-containing conditions. These results are consistent with those of a previous report (17).

Notably, 2I system is sufficient for the robust proliferation of mESCs in serum-free culture conditions (Fig. 1, *E*–*J*) (9). To investigate whether Go6983 has the same effect, mESCs at a low density were cultured in N2B27 medium. After one passage, the Go6983-treated mESCs maintained an undifferentiated state, whereas the NT group cells began to die (Fig. 1*G*). Moreover, mESCs could be split for 10 more passages and grown under N2B27/Go6983 conditions (Fig. 1, *G* and *H*). These cells exhibited high levels of the pluripotency gene Sox2 (Fig. 1, *I* and *J*). To further explore the differentiation capacity of these cells, they were grown in suspension to form embryoid bodies (EBs). After 8 days, the EBs were dissociated and seeded on gelatin-coated plates. Tuj1^+^ neurons, myosin^+^ myocardial cells, and Gata4^+^ primitive endoderm cells were observed (Fig. 1*K*). However, Go6983-treated mESCs eventually collapsed and lost their undifferentiated state. These data suggest that the inhibition of PKC by Go6983 is able to support short-term mESC self-renewal in N2B27 medium.

### Go6983 represses *de novo* methyltransferase activity and induces global DNA demethylation

**Figure 2:**
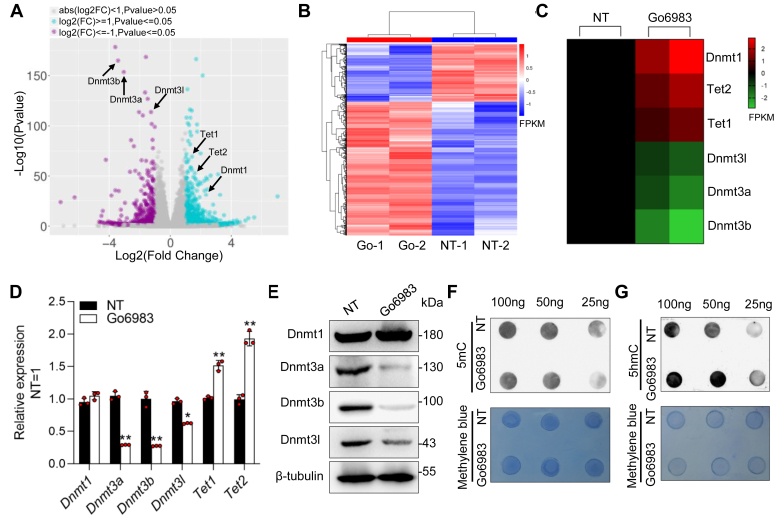
**Go6983 represses *de novo* methyltransferases activity and induces global DNA demethylation.***A*, volcano map showing the differentially expressed genes in mESCs regulated by Go6983. The values on the x-axis are the log2-fold changes in the transcript levels (fold change = FPKM value of genes in the Go6983 treatment/FPKM value of genes in the NT control). *B*, heatmap showing the gene expression patterns regulated by Go6983 in mESCs. The genes were arranged according to the value of FPKM. The *red* to *black* to *green* colors represent FPKM values from high to low, as shown in the scale bar. Two replicates were performed for each sample. *C*, heatmaps showed the expression levels of *Dnmt1, Dnmt3a, Dnmt3b, Dnmt3l, Tet1*, and *Tet2*. The genes were arranged according to the value of FPKM value. Two replicates were performed for each sample. *D*, qRT‒PCR analysis of *Dnmt1, Dnmt3a, Dnmt3b, Dnmt3l, Tet1*, and *Tet2* expression in mESCs treated with or without Go6983 for 24 h. Data represented as the mean ± SD (N = 3 biological replicates). ∗*p* < 0.05, ∗∗*p* < 0.01 *versus* NT, as determined by two paired Student’s *t* test. *E*, Western blot analysis of Dnmt1, Dnmt3a, Dnmt3b, and Dnmt3l levels in mESCs in the presence or absence of Go6983 for 24 h. β-tubulin was used as a loading control. *F*, dot blotting analysis of 5mC levels in 25 ng, 50 ng, and 100 ng of DNA in the presence or absence of Go6983 for 24 h. Methylene blue staining was used to validate equal amounts of DNA loaded. *G*, dot blotting analysis of 5hmC levels in 25 ng, 50 ng, and 100 ng of DNA from mESCs treated with or without Go6983 for 24 h. FPKM, Fragment Per Kilobase of exon model per Million mapped reads; mESCs, mouse embryonic stem cells; NT, no treatment; qRT-PCR, quantitative real-time PCR.

To gain insight into the mechanism by which Go6983 promotes mESC self-renewal, we compared the global transcription profiles of NT cells and Go6983-treated cells by RNA-sequencing (GSE185220). We identified 1100 upregulated genes and 478 downregulated genes that were regulated more than 2-fold in the Go6983-treated cells compared to the NT cells (Fig. 2, *A* and *B*). Notably, the epigenetic regulators Dnmts and Tets attracted our attention because they cooperate to regulate the promoter epigenetic landscapes of ESCs and are closely associated with ESC maintenance (28). Specifically, two *de novo* methyltransferases, *Dnmt3a* and *Dnmt3b*, and their regulator *Dnmt3l* were decreased, whereas the expression of the DNA methyltransferase *Dnmt1* was unchanged (Fig. 2*C*). In contrast, the expression levels of the DNA hydroxylase *Tet1* and *Tet2* were increased slightly (Fig. 2*C*). Subsequently, quantitative real-time PCR (qRT‒PCR) was used to validate the expression of the *Dnmt3* and *Tet* genes. In agreement with the sequencing analysis, the expression of the genes encoding *Dnmt3a, Dnmt3b*, and *Dnmt3l* decreased, while the expression of *Tet1* and *Tet2* increased (Fig. 2*D*). The decrease in the protein levels of Dnmt3 members induced by Go6983 was also confirmed by Western blot analysis (Fig. 2*E*). Therefore, we investigated whether Go6983 results in genome-wide erasure of DNA methylation, and the global levels of 5-methylcytosine (5mC) and 5-hydroxymethylcytosine(5hmC) in three different concentrations of total DNA were detected *via* the Dot blot method. As expected, compared with that in NT cells, the global 5mC content in Go6983-treated cells was decreased, but the 5hmC level was increased (Fig. 2, *F* and *G*). These data suggest that the cumulative effect of *Dnmt3* downregulation may be responsible for the changes in global DNA methylation observed upon Go6983 treatment.

### Go6983 triggers Prdm14 transcription to suppress Dnmt3 gene expression

**Figure 3:**
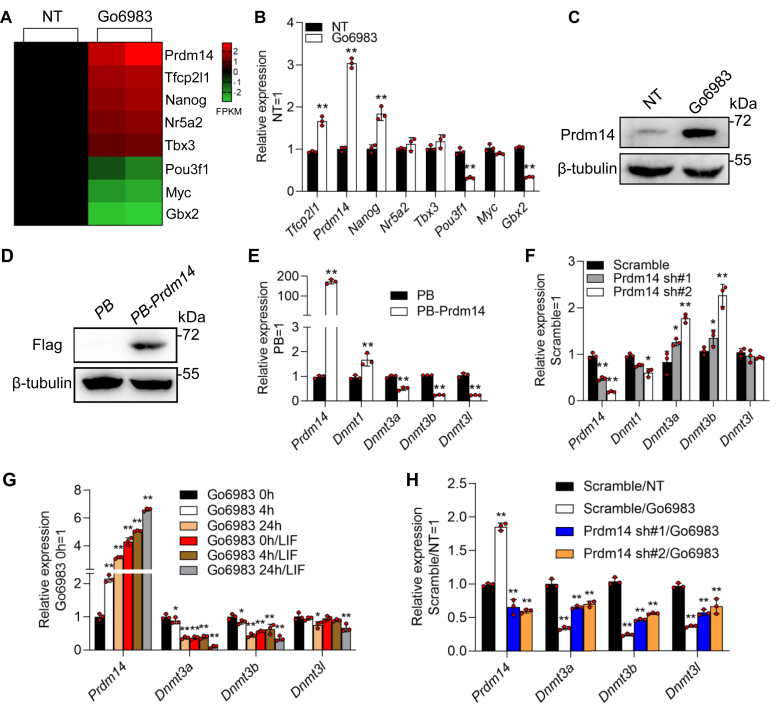
**Go6983 triggers Prdm14 transcription to suppress Dnmt3 gene expression**. *A*, the heatmap shows the expression patterns of stem cell pluripotency-associated genes regulated by Go6983. Genes were ranked according to the FPKM value. The *red* to *black* to *green* colors represent FPKM values from high to low, as shown in the scale bar. Two replicates were performed for each sample. *B*, qRT‒PCR analysis of the expression levels of *Prdm14, Tfcp2l1, Nanog, Nr5a2, Tbx3, Pou3f1, Myc*, and *Gbx2* in 46C mESCs treated with or without Go6983 for 24 h. Data represented as the mean ± SD (N = 3 biological replicates). ∗*p* < 0.05, ∗∗*p* < 0.01 *versus* NT, as determined by two paired Student’s *t* test. *C*, Western blot analysis of Prdm14 protein levels in 46C mESCs treated with or without Go6983 for 24 h. *D*, Western blot analysis of Flag in 46C mESCs overexpressing the Flag-tagged *Prdm14* gene. *E*, qRT‒PCR analysis of the expression levels of *Prdm14, Dnmt1, Dnmt3a, Dnmt3b*, and *Dnmt3l* in *PB-* and *PB-Prdm14*-expressing 46C mESCs in the absence of LIF. Data represented as the mean ± SD (N = 3 biological replicates). ∗*p* < 0.05, ∗∗*p* < 0.01 *versus PB*, as determined by two paired Student’s *t* test. *F*, qRT‒PCR analysis of *Prdm14, Dnmt1, Dnmt3a, Dnmt3b*, and *Dnmt3l* in s*crambled* shRNA- and *Prdm14* shRNA-expressing mESCs. Data represented as the mean ± SD (N = 3 biological replicates). ∗*p* < 0.05, ∗∗*p* < 0.01 *versus Scramble*, as determined by one-way ANOVA with Sidak’s multiple comparisons test. *G*, qRT‒PCR analysis of the expression of *Prdm14, Dnmt3a, Dnmt3b*, and *Dnmt3l* in 46C mESCs treated with Go6983 for 4 or 24 h. Data represented as the mean ± SD (N = 3 biological replicates). ∗*p* < 0.05, ∗∗*p* < 0.01 *versus* Go6983 0h, as determined by one-way ANOVA with Sidak’s multiple comparisons test. *H*, qRT‒PCR analysis of the expression of *Prdm14, Dnmt3a, Dnmt3b*, and *Dnmt3l* in *scrambled* shRNA- and *Prdm14* shRNA-expressing mESCs maintained in the presence or absence of Go6983. Data represented as the mean ± SD (N = 3 biological replicates). ∗*p* < 0.05, ∗∗*p* < 0.01 *versus Scramble*/NT, as determined by one-way ANOVA with Sidak’s multiple comparisons test. FPKM, Fragment Per Kilobase of exon model per Million mapped reads; LIF, leukemia inhibitory factor; mESCs, mouse embryonic stem cells; NT, no treatment; qRT-PCR, quantitative real-time PCR.

To elucidate the molecular basis of the Go6983-mediated *Dnmt3* gene repression, we examined the differently expressed genes revealed by RNA-sequencing to be enriched in signaling pathways regulating the pluripotency of stem cells *via* the Kyoto Encyclopedia of Genes and Genomes method. A short list of eight candidates emerged, including *Tfcp2l1, Prdm14, Nanog, Nr5a2, Tbx3, Pou3f1, Myc*, and *Gbx2* (Figs. 3*A* and S1, *A* and *B*). We confirmed these findings by qRT‒PCR (Fig. 3*B*). Among these genes, *Prdm14* attracted our attention because constitutive expression of *Prdm14* represses *de novo* DNA methylation of pluripotency-associated genes (29, 30, 31, 32). Moreover, the upregulation of the *Prdm14* protein was further validated by Western blotting (Fig. 3*C*). We therefore speculated that Prdm14 may act as an intermediate bridge to transduce the signaling of Go6983 to *Dnmt3* genes to reduce DNA methylation. To test this hypothesis, we designed four approaches. First, we generated an mESC line that overexpressed *FLAG*-tagged *Prdm14* using a PiggyBac vector (*PB-Prdm14*), in which *Prdm14* expression was efficiently enhanced (Fig. 3*D*). As expected, enforced expression of *Prdm14* significantly inhibited the expression of *Dnmt3a, Dnmt3b*, and *Dnmt3l* (Fig. 3*E*). Second, 46C mESCs were infected with lentiviruses encoding two shRNAs specific for *Prdm14* mRNA (*Prdm14* sh#1 and *Prdm14* sh#2). Stable knockdown (80–90%) of *Prdm14* transcript levels was observed following drug selection (Fig. 3*F*). As a result, the transcript levels of *Dnmt1, Dnmt3a*, and *Dnmt3b* but not *Dnmt3l* increased in *Prdm14* shRNA cells compared with those in *scramble* control mESCs (Fig. 3*F*). Third, 46C mESCs were treated with Go6983 for different durations (4 h and 24 h) in the presence or absence of LIF. As shown in Figure 3*G*, the expression of the *Prdm14* and *Dnmt3* genes exhibited opposite patterns (Fig. 3*G*). Finally, *scrambled* shRNA- and *Prdm14* shRNA-expressing 46C mESCs were treated with Go6983, and the results showed that knockdown of *Prdm14* inhibited the repressive effect of Go6983 on the *Dnmt3* genes, although it did not completely restore *Dnmt3* transcription (Fig. 3*H*). Taken together, these data suggest that Go6983 limits *Dnmt3* gene expression partially through upregulation of *Prdm14* expression.

### The addition of Go6983 suppressed Dnmt3 gene expression mainly *via* the inhibition of PKCδ activity

**Figure 4:**
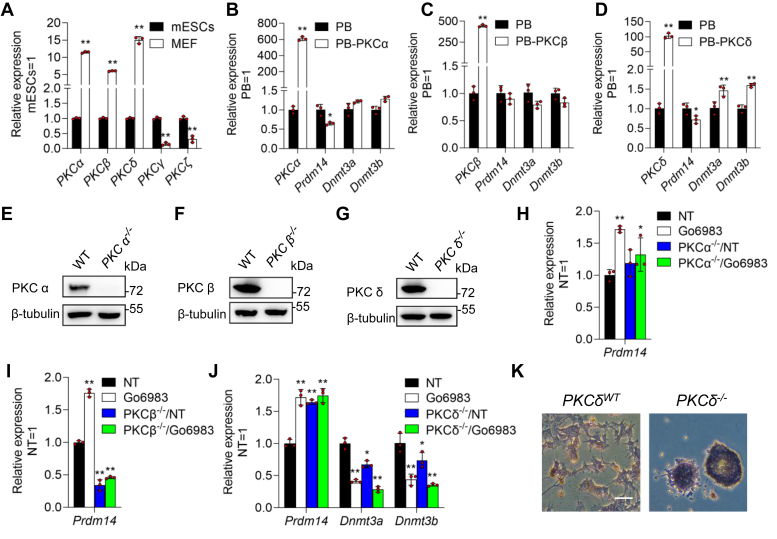
**The effects of different PKC isoforms on Prdm14 expression**. *A*, qRT‒PCR analysis of the expression levels of *PKCα, β, δ, γ*, and *ζ* in 46C mESCs and mouse embryonic fibroblasts. Data represented as the mean ± SD (N = 3 biological replicates). ∗*p* < 0.05, ∗∗*p* < 0.01 *versus* mESCs, as determined by two paired Student’s *t* test. *B–D*, qRT‒PCR analysis of the expression levels of the *PKC* isoforms *Prdm14, Dnmt3a* and *Dnmt3b* in *PB, PB-PKCα, PB- PKCβ*, or *PB-PKCδ* transfected 46C mESCs in the absence of LIF. Data represented as the mean ± SD (N = 3 biological replicates). ∗*p* < 0.05, ∗∗*p* < 0.01 *versus PB*, as determined by two paired Student’s *t* test. *E*–*G*, Western blot analysis of the protein levels of PKCα, PKCβ, and PKCδ in WT and *PKCα*^*−/−*^, *PKCβ*^*−/−*^, and *PKCδ*^*−/−*^ mESCs. *H*–*J*, qRT‒PCR analysis of the expression of *Prdm14, Dnmt3a* or *Dnmt3b* in WT, *PKCα*^*−/−*^, *PKCβ*^*−/−*^, and *PKCδ*^*−/−*^ mESCs maintained in the presence or absence of Go6983. Data represented as the mean ± SD (N = 3 biological replicates). ∗*p* < 0.05, ∗∗*p* < 0.01 *versus* NT, as determined by one-way ANOVA with Sidak’s multiple comparisons test. *K*, AP staining of WT and *PKCδ*^*−/−*^ mESCs cultured in the absence of LIF for 8 days. Scale bar, 100 μm. AP, alkaline phosphatase; LIF, leukemia inhibitory factor; mESCs, mouse embryonic stem cells; NT, no treatment; PKC, protein kinase C; qRT-PCR, quantitative real-time PCR; WT, wildtype.

Several members of the PKC family, including PKCα, PKCβ, PKCγ, PKCδ, and PKCζ, exhibit different expression patterns in differentiated and undifferentiated cells. Compared to mouse embryonic fibroblasts, 46C mESCs harbor higher levels of *PKCγ* and PKC*ζ* but lower levels of *PKCα*, PKC*β*, and PKC*δ* (Fig. 4*A*), suggesting that PKCα, PKCβ, and PKCδ may be associated with mESC differentiation. Next, we investigated which of these genes is responsible for the induction of *Prdm14*. First, the coding sequences of *PKCα*, PKC*β*, and PKC*δ* were inserted into the PB system. All the transfectants were maintained in LIF/serum medium. We found that *PKCα* and *PKCδ* repressed *Prdm14* transcription but increased the expression of *Dnmt3a* and *Dnmt3b* (Fig. 4, *B*–*D*). Subsequently, three PKC family members were deleted in mESCs *via* the CRISPR/Cas9 system (Fig. 4, *E*–*G*). Only the knockout of *PKCδ* was capable of inducing *Prdm14* expression (Fig. 4, *H*–*J*). Importantly, Go6983 failed to increase the *Prdm14* transcript in the absence of *PKCδ* (Fig. 4*J*). In addition, *PKCδ* knockout mESCs could sustain an undifferentiated state when seeded at a low density without LIF treatment for 8 days (Fig. 4*K*). Overall, these data indicate that Go6983 induces *Prdm14* mainly by inhibiting the activity of PKCδ.

### Prdm14 mediates the self-renewal–promoting effect of Go6983

**Figure 5:**
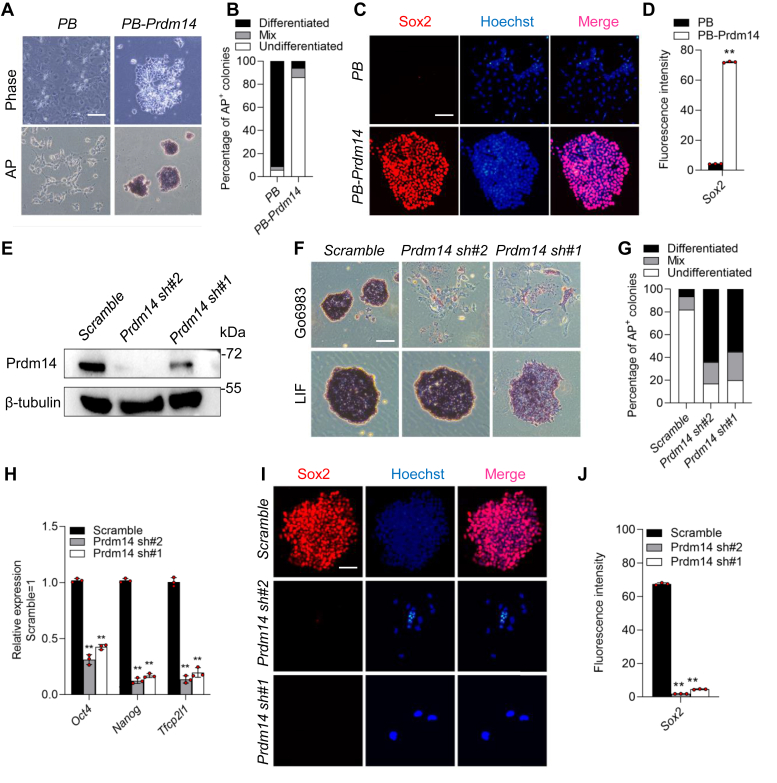
**Prdm14 mediates the self-renewal-promoting effect of Go6983.***A*, morphology and AP staining of *PB-* and *PB-Prdm14-*expressing 46C mESCs cultured in serum-containing medium in the absence of LIF for 8 days. Scale bar, 100 μm. *B*, quantification of AP-positive colonies in (*A*). *C*, immunofluorescence staining of Sox2 in *PB* and *PB-Prdm14* mESCs in the absence of LIF for 8 days. Scale bar, 100 μm. *D*, quantification of fluorescence intensity (*red*) in (*B*). Data represented as the mean ± SD (N = 3 biological replicates). ∗∗*p* < 0.01 *versus* PB, as determined by two paired Student’s *t* test. *E*, Western blot analysis of Prdm14 protein levels in 46C mESCs infected with *scrambled* shRNA or *Prdm14* shRNA lentivirus. *F*, AP staining of *scrambled* shRNA- and *Prdm14* shRNA-expressing cells cultured in serum-containing medium supplemented with LIF or Go6983 for 8 days. Scale bar, 100 μm. *G*, quantification of AP-positive colonies in (*F*). *H*, qRT‒PCR analysis of the expression of the pluripotency genes *Oct4, Nanog*, and *Tfcp2l1* in s*crambled shRNA-*and *Prdm14* shRNA-expressing 46C mESCs treated with or without Go6983. Data represented as the mean ± SD (N = 3 biological replicates). ∗∗*p* < 0.01 *versus Scramble*, as determined by one-way ANOVA with Sidak’s multiple comparisons test. *I*, immunofluorescence analysis of Sox2 in *scrambled* shRNA- and *Prdm14* shRNA-expressing cells in the presence of Go6983 for 8 days. Scale bar, 100 μm. *J*, quantification of fluorescence intensity (*red*) in (*I*). Data represented as the mean ± SD (N = 3 biological replicates). ∗∗*p* < 0.01 *versus Scramble*, as determined by one-way ANOVA with Sidak’s multiple comparisons test. AP, alkaline phosphatase; LIF, leukemia inhibitory factor; mESCs, mouse embryonic stem cells; qRT-PCR, quantitative real-time PCR.

To functionally investigate whether *Prdm14* has the ability to phenocopy the function of Go6983 in mESCs, *PB* empty vector controls and *PB-Prdm14* mESCs were seeded in serum/LIF-containing medium at the same low density. After LIF withdrawal, the *PB-Prdm14* mESCs remained undifferentiated after 8 days (Fig. 5, *A* and *B*). These transfectants exhibited increased AP activity and Sox2 expression (Fig. 5, *A*–*D*). However, the *PB* control cells were completely differentiated (Fig. 5, *A*–*D*). These data indicate that ectopically expressed *Prdm14* is capable of recapitulating the effect of Go6983 on mESC clonogenicity.

To determine whether Go6983 depends on *Prdm14* to maintain the undifferentiated state of mESCs, we validated the downregulation of the *Prdm14* protein in 46C mESCs by Western blot analysis (Fig. 5*E*). The *scramble* control and *Prdm14* shRNA cells were well maintained under LIF/serum conditions (Fig. 5, *F* and *G*), suggesting that *Prdm14* maintains pluripotency independently of LIF/Stat3. However, knockdown of *Prdm14* induced mESC differentiation in the presence of Go6983, as indicated by the flat cell morphology, decreased AP activity, and low expression levels of the pluripotency markers *Sox2, Oct4, Nanog*, and *Tfcp2l1* (Fig. 5, *F*–*J*), suggesting that downregulation of *Prdm14* impairs the Go6983-mediated mESC self-renewal but is dispensable for the effect of LIF.

### Prdm14 is critical for Go6983-mediated promotion of human-induced pluripotent stem cell self-renewal

As the inhibition of pan-PKC with another selective inhibitor, GF109203X (GFX), has been shown to facilitate the maintenance of human-induced pluripotent stem cells (hiPSCs) (19), we first wanted to determine the effect of Go6983 on hiPSC stemness. hiPSCs cultured in basal medium quickly differentiated after one passage, whereas Go6983-treated hiPSCs were split into three additional passages and stained positive for Sox2 but subsequently collapsed and lost AP activity (Fig. S2, *A* and *B*). Therefore, Go6983 can support only the short-term self-renewal of hiPSCs. Next, to examine whether Go6983 can induce *Prdm14* expression, qRT‒PCR was carried out to detect *Prdm14* transcripts after the hiPSCs were treated with Go6983 for 24 h. As shown in Fig. S2*C*, compared with those in the NT control cells, the *Prdm14* levels were increased (Fig. S2*C*). Moreover, the addition of Go6983 repressed *Dnmt3* gene expression (Fig. S2*D*). Finally, we knocked down human *Prdm14* in hiPSCs infected with two *Prdm14* shRNA lentiviruses (Fig. S2*E*). After two passages, we found that *Prdm14* shRNA caused an obvious reduction in the ability to form undifferentiated colonies in the presence of Go6983 (Fig. S2, *F* and *G*). These findings highlight the important role of *Prdm14* in mediating the function of Go6983 in hiPSCs.

Conversely, to investigate whether GFX has a function similar to that of Go6983 in mESCs, 46C mESCs were seeded in serum-containing medium supplemented with different concentrations of GFX. After 8 days, we observed that AP-positive stem cell colonies developed in a dose-dependent manner (Fig. S3*A*), whereas 15 μM GFX caused growth arrest and cell death (Fig. S3*A*). GFX induced *Prdm14* expression (Fig. S3, *B* and *C*). Similarly, GFX repressed *Dnmt3a* and *Dnmt3b* expression and increased *Tet2* transcription (Fig. S3*D*). Additionally, knockdown of *Prdm14* eliminated the self-renewal-promoting effect of GFX (Fig. S3, *E* and *F*). Taken together, these results indicate that *Prdm14* is important for GFX function in mESCs.

### Go6983 enhances the expression of Prdm14 by alleviating Suv39h-mediated dimethylation and trimethylation of H3K9

**Figure 6:**
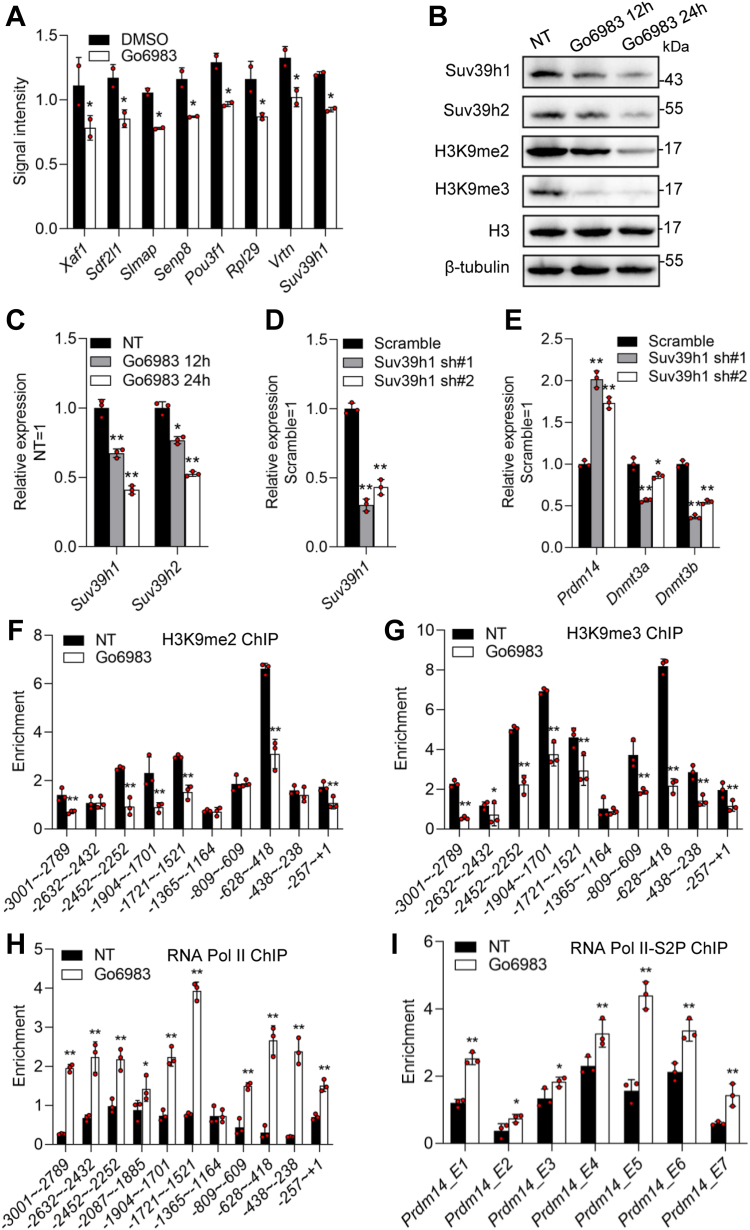
**Go6983 enhances the expression of Prdm14 *via* alleviating Suv39h-mediated dimethylation and trimethylation of H3K9**. *A*, LC‒MS was used to perform unbiased quantitative analysis of the signal intensity of the indicated proteins in 46C mESCs treated with or without Go6983 for 24 h. The data are presented as the mean ± SD (N = 2 biological replicates). ∗*p* < 0.05 *versus* DMSO, as determined by two paired Student’s *t* test. *B*, Western blot analysis of the levels of Suv39h1, Suv39h2, H3, H3K9me2, and H3K9me3 in 46C mESCs cultured in the presence of Go6983 for 12 h or 24 h. *C*, qRT‒PCR analysis of *Suv39h1* and *Suv39h2* expression in 46C mESCs treated with Go6983 for 12 h or 24 h. Data represented as the mean ± SD (N = 3 biological replicates). ∗*p* < 0.05, ∗∗*p* < 0.01 *versus* NT, as determined by one-way ANOVA with Sidak’s multiple comparisons test. *D*, qRT‒PCR analysis of *Suv39h1* transcripts in 46C mESCs infected with *scrambled* shRNA or *Suv39h1* shRNA lentivirus. Data represented as the mean ± SD (N = 3 biological replicates). ∗*p* < 0.05, ∗∗*p* < 0.01 *versus Scramble*, as determined by one-way ANOVA with Sidak’s multiple comparisons test. *E*, qRT‒PCR analysis of the expression levels of *Prdm14, Dnmt3a*, and *Dnmt3b* in *scrambled* shRNA*-* and *Suv39h1* shRNA-expressing mESCs. Data represented as the mean ± SD (N = 3 biological replicates). ∗*p* < 0.05, ∗∗*p* < 0.01 *versus Scramble*, as determined by one-way ANOVA with Sidak’s multiple comparisons test. *F,G*, qRT‒PCR analysis of the enrichment of H3K9me2 and H3K9me3 in the different regions of the Prdm14 promoter. Data represented as the mean ± SD (N = 3 biological replicates). ∗*p* < 0.05, ∗∗*p* < 0.01 *versus* NT, as determined by two paired Student’s *t* test. *H*, following a ChIP assay in which an antibody was used to detect RNA polymerase II in mESCs treated with or without Go6983 for 24 h, the promoter fragments of *Prdm14* were amplified. Data represented as the mean ± SD (N = 3 biological replicates). ∗*p* < 0.05, ∗∗*p* < 0.01 *versus* NT, as determined by two paired Student’s *t* test. *I*, following a ChIP assay using an antibody to detect the Ser2 phosphorylated form of the RNA polymerase II CTD in mESCs treated with or without Go6983 for 24 h, the results were analyzed as described in (*H*), except that the primers used the coding region but not the promoters as template. Data represented as the mean ± SD (N = 3 biological replicates). ∗*p* < 0.05, ∗∗*p* < 0.01 *versus* NT. E1: the first pair of primers for amplification of the fragment of the exon, as determined by two paired Student’s *t* test. ChIP, chromatin immunoprecipitation; CTD, C-terminal domain; mESCs, mouse embryonic stem cells; NT, no treatment; qRT-PCR, quantitative real-time PCR.

To elucidate how Go6983 induces *Prdm14* transcription, we used iTRAQ-coupled liquid chromatography–mass spectrometry to perform an unbiased quantitative analysis of the protein profile of 46C mESCs treated with or without Go6983 for 24 h. Compared to those in the DMSO-treated control group, 28 proteins were differentially expressed in the Go6983-treated cells; the protein levels of 20 genes were upregulated by 1.3-fold or more, while the protein levels of eight genes were downregulated -by more than 20%. These proteins included Suv39h1, Vrtn, Rpl29, Pou3f1, Senp8, Slmap, Sdf2f1, and Xaf1 (Fig. 6*A* and Table S1). Subsequently, we focused on Suv39h1 because it is a well-known transcriptional repressor that promotes dimethylated or trimethylated H3K9 (H3K9me2/3). To examine whether the addition of Go6983 increases *Prdm14 via* Suv39h1, three approaches were used. First, the expression of Suv39h1 and its family member Suv39h2 was assessed following Go6983 treatment. In addition, both Suv39h1 and Suv39h2 decreased, but the control proteins Ezh1 and Ezh2, which are responsible for the trimethylation of H3K27 (H3K27me3) and are linked to ESC identity and pluripotency (33), did not change (Figs. 6, *B* and *C* and S4*A*). Consistent with these Western blot data, there was a concomitant reduction in H3K9me2 and H3K9me3, but not H3K27me3, Ezh1, Ezh2, and total H3 protein upon Go6983 treatment (Figs. 6, *B* and *C* and S4*A*). Second, RNA interference was carried out to repress *Suv39h1* transcription (Fig. 6*D*). Downregulation of *Suv39h1* enhanced *Prdm14* expression but inhibited *Dnmt3a* and *Dnmt3b* expression (Fig. 6*E*). In line with these results, chaetocin, a selective inhibitor of Suv39h1, was applied. The addition of chaetocin induced *Prdm14* expression but not PF-06726304, a specific inhibitor of Ezh2 (Fig. S4, *B* and *C*). Notably, overexpression of *Prdm14* did not change the levels of Suv39h1, Suv3h2, H3K9me2, and H3K9me3 (Fig. S4*D*). Third, chromatin immunoprecipitation (ChIP)–qRT‒PCR was performed for H3K9me2 and H3K9me3 at the promoter region of Prdm14. A series of primer pairs was thus designed to interrogate this region. As expected, there was an obvious decrease in H3K9me2 and H3K9me3 at the *Prdm14* promoter in Go6983-treated mESCs compared to those in control ESCs (Fig. 6, *F* and *G*). Finally, we investigated whether PKC inhibition affects the recruitment of RNA polymerase II to target genes. ChIP assay revealed that Go6983 treatment led to an increase in total RNA polymerase II levels at these promoters (Fig. 6*H*). Furthermore, a ChIP study of Ser2 phosphorylation of the C-terminal domain repeats of RNA polymerase II (RNA Pol II–S2P) showed that the addition of Go6983 led to increased enrichment of the elongating form of RNA Pol II–S2P in the gene bodies of *Prdm14* (Fig. 6*I*). Taken together, these results indicate that the inhibition of PKC reduces the levels of the repressive histone marks H3K9me2 and H3K9me3 at the promoter of *Prdm14* by decreasing the Suv39h1 protein level, which thereby stimulates *Prdm14* expression.

## Discussion

**Figure 7:**
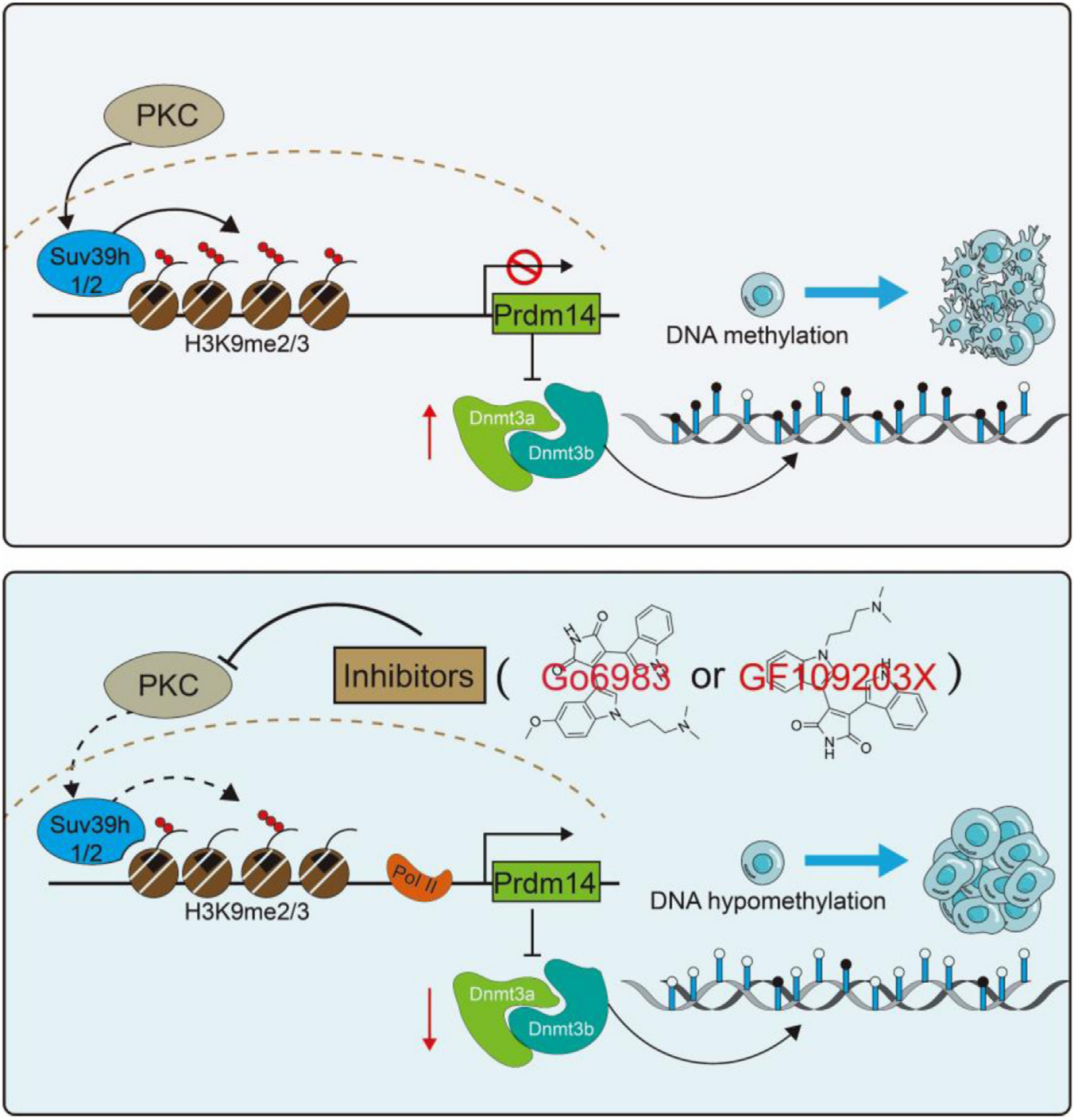
**Diagram of the working mode of PKC inhibitors in ESCs.** Schematic diagram of PKC inhibitors input to the molecular circuitry underlying the pluripotency of ESCs. The PKC inhibitors Go6983 and GF109203X reduced the protein levels of Suv39h1 and Suv39h2, resulting in the downregulation of H3K9me2 and H3K9me3. RNA polymerase Ⅱ was subsequently recruited to induce *Prdm14* transcription and thereby repress the expression of *Dnmt3a* and *Dnmt3b*. Hypomethylation of DNA is beneficial for maintenance of ESCs. ESCs, embryonic stem cells; PKC, Protein kinase C.

Inhibition of PKC plays an important role in promoting self-renewal of ESCs, but the downstream molecular mechanism involved remains unclear. Through RNA-sequencing, we found that the PKC inhibitor Go6983 could maintain the hypomethylation state of mESCs by inhibiting the expression of the *Dnmt3* genes. Functional studies further showed that Go6983 suppresses the transcription of *Dnmt3* genes partially by inducing the expression of the *Prdm14* gene. Therefore, knocking down the expression of the *Prdm14* gene effectively inhibited the effect of Go6983 on maintaining the undifferentiated state of mESCs. A proteomic profile further showed that the inhibition of PKC reduced the epigenetic enzyme Suv39h-mediated H3K9me2/3 and thereby facilitated *Prdm14* transcription (Fig. 7). These findings expand our understanding of the regulatory networks involved in stem cell pluripotency.

DNA methylation plays an important role in the control of gene transcription during the early development of mammals, and its patterns change dynamically (34). Previous studies have demonstrated that Dnmt3a and Dnmt3b, in combination with the catalytically inactive cofactor Dnmt3l, determine DNA methylation patterns during embryogenesis, and these patterns are maintained by Dnmt1 (35, 36). Accumulating evidence indicates that culture conditions can significantly affect DNA methylation patterns in pluripotent cell lines (32, 37, 38, 39). Whole-genome bisulfite sequencing revealed that mESC lines grown in conventional culture media serum/LIF had DNA methylation profiles that resembled those of postimplantation epiblasts, while 2I stalls mESCs in a hypomethylated, ICM-like state (32, 37, 38). Therefore, 2I-cultured mESC populations acquire a more naïve pluripotent state. 2I induced demethylation through direct repression of the *de novo* methyltransferase genes *Dnmt3a* and *Dnmt3b*, as well as upregulation of the protein levels of *Tet1* and *Tet2* (32, 37, 38, 39). The global hypomethylation status ensures the activation of pluripotency-associated genes. Our results also showed that inhibition of pan-PKC can induce a hypomethylated state in mESCs with low expression levels of *Dnmt3a* and *Dnmt3b* (Fig. 2, *B*–*E*). Therefore, the application of a PKC inhibitor is sufficient to derive germline-competent mESCs as demonstrated by the observation of a naïve pluripotent state sustained by Go6983 (17). This may also partly explain why the inhibition of PKC is beneficial for inducing naïve pluripotency in human ESCs (40, 41), as global inhibition of Dnmt activity was shown to facilitate the reprogramming of differentiated somatic cells to pluripotency (42). However, hypomethylation may not be the only downstream mechanism of Go6983 because *Dnmt*-null ESCs can be readily derived from blastocysts or by direct gene targeting even in the case of triple knockouts of all catalytically active Dnmts (Dnmt1, 3a, and 3b) (43).

Another important finding of our study was that Prdm14 is critical for the self-renewal-promoting effect of Go6983 (Fig. 5, *E*–*J*). As an important bridge, this is reasonable. First, *Prdm14* is transiently expressed in the inner cell mass of the blastocyst at embryonic Day 3.5, followed by rapid downregulation in the epiblast at the postimplantation stage in mice (44). Second, mESCs cannot be derived from *Prdm14*-null blastocysts under serum conditions (29). A previous study showed that Prdm14 prevents the induction of extraembryonic endoderm differentiation in EBs (45). Ectopic expression of *Prdm14* thus maintains long-term mESC pluripotency and self-renewal after the withdrawal of the LIF (31). In humans, *Prdm14*, which is expressed specifically in undifferentiated human ESCs and suppresses the transcription of differentiation genes in EBs, was identified as one of the major determinants of human ESC identity (46, 47). However, depletion of *Prdm14* results in an increase in the expression of developmental genes (46, 47). Third, Prdm14 directly binds to the upstream region of *Dnmt3a/b/l* and represses their transcription (29, 30, 32). In *Prdm14*-deficient ESCs, DNA methylation levels and *Dnmt3a/b/l* expression are consistently maintained at high levels (29). Therefore, Prdm14 is responsible for global hypomethylation through transcriptional repression of *Dnmt3* genes in naïve pluripotent state of ESCs. DNA demethylation can occur through passive or active mechanisms. Passive DNA demethylation is induced by suppressing the activity of DNA methyltransferases, while active DNA demethylation is triggered by Tet proteins through hydroxylation of 5mC to 5hmC (48). We observed that Go6983 increased *Tet1* and *Tet2* levels (Fig. 2, *C* and *D*). In addition, Prdm14 physically interacts with Tet1 and Tet2 and enhances their recruitment to target loci to achieve transcriptional regulation and DNA demethylation (30). Notably, Prdm14 is also a critical germ cell regulator that plays a role in the generation of primordial germ cells (44). It will be of great interest in future studies to determine whether PKC inhibitors impact the primordial germ cell specification.

Methylation of histone lysine residues can affect the structure of chromatin regions and usually functions as a crucial epigenetic mechanism to regulate gene expression. For instance, the formation of heterochromatin is catalyzed by the histone methyltransferase Suv39h1 and the closely related enzyme Suv39h2, which are the principal enzymes responsible for the accumulation of histone H3, which contains a dimethylated and trimethylated at the lysine 9 position (H3K9me2 and H3K9me3, respectively) (49). In turn, these histone marks recruit heterochromatin protein 1 to mediate downstream silencing of the affected chromatin domains (50, 51). H3K9 histone methyltransferases are closely involved in the maintenance and induction of stem cell pluripotency. Bernard *et al*. reported that Oct4 can activate the expression of *Suv39h1as*, an antisense long noncoding RNA to Suv39h1, to downregulate *Suv39h1* transcription and H3K9me2 and H3K9me3 levels in mESCs, and these results reveal how global H3K9 methylation is regulated at the onset of differentiation to irreversibly exit pluripotency (52). In addition, H3K9 methylation is the primary epigenetic determinant of prepluripotent stem cells, and its removal leads to fully reprogrammed pluripotent stem cells (53). In addition to histone methyltransferases, histone demethylases, Jmjd1a and Jmjd2c, also play important roles in sustaining self-renewal genes in mESCs (54). Jmjd1a demethylates H3K9me2 at the promoter regions of *Tcl1, Tcfcp2l1*, and *Zfp57* and positively regulates their expression, while Jmjd2c acts as a positive regulator of *Nanog* by reversing the H3K9me3 marks at its promoter to prevent heterochromatin protein 1 from binding (54). Indeed, a PKC inhibitor can also enhance Jmjd2d and Jmjd3 expression to decrease H3K9me3 signaling at transcription start sites of self-renewal genes in mESCs (55). Our results revealed that Go6983 inhibits the expression of *Suv39h1* and *Suv39h2* (Fig. 6, *B* and *C*). Overall, these data indicate that both histone methylases and demethylases are involved in the self-renewal-promoting effect of PKC inhibition.

However, how Go6983 negatively regulates the expression of Suv39h genes remains unclear. Moreover, a more detailed understanding of the recruitment and target genes of Suv39h genes in maintaining ESC identity will enable a better understanding of the role of PKC inhibitors. On the other hand, in addition to H3K9 and DNA methylation, the inhibition of PKC also regulates other epigenetic mechanisms, such as H3K27 trimethylation, nucleosome remodeling, and deacetylation. However, how PKC inhibition coordinates and balances their functions in modulating the expression of self-renewal- and differentiation-associated genes still needs further investigation (55, 56).

In summary, we provide detailed evidence that the PKC inhibitor Go6983 promotes *Prdm14/Dnmt*-mediated passive DNA demethylation partially by reducing Suv39h-mediated H3K9me2/3 modification. However, the function of PKC inhibitors appears to be conserved between mouse and human pluripotent stem cells as revealed by the findings of this and other studies (17, 19). However, there are many characteristic differences between the two species and the corresponding ESCs (1). A precise understanding of the transcriptional dynamics and epigenetic characteristics of Go6983 will shed new light on the regulation of different pluripotent states.

## Experimental procedures

### Cell culture

46C mESCs were cultured in 0.1% gelatin-coated dishes at 37 °C in an incubator supplemented with 5% carbon dioxide. mESCs were routinely maintained in Dulbecco's modified Eagle's medium (2310048, ViVa Cell Biosciences) supplemented with 15% FBS (FND500, ExCell Bio), 1 × MEM nonessential amino acids (11140050, Gibco), 0.1 mM β-mercaptoethanol (M3148, Sigma), and 1000 U/ml LIF (Made in house). hiPSCs were maintained on plates precoated with Matrigel (BD Biosciences) at 37 °C under 5% CO_2_. The basal medium of the hiPSCs was composed of N2B27, 10% KSR (Invitrogen), 10 ng/ml activin A (PeproTech), and 10 ng/ml bFGF (PeproTech). Y27632(1 mM, HY-10583, MedChem Express) was added to the culture medium after the cells were passaged. All cell lines were free from *mycoplasma* contamination for all experiments.

### Plasmid construction

The coding regions of the mouse genes *Prdm14, PKCα, PKCβ*, and *PKCδ* were inserted into PiggyBac transposon vectors. The targeting sequences designed for decreasing *Prdm14* and *Suv39h1* transcripts were cloned and inserted into pLKO.1-TRC vector (#10878, Addgene). The *PKCα, PKCβ*, and *PKCδ* knockout sequences were subsequently cloned and inserted into pX330-U6-Chimeric_BB-CBh-hSpCas9 (Addgene) The sequences are listed in Tables S2–S4.

### AP activity assay

The AP activity of ESCs was detected using an Alkaline Phosphatase Kit (C3206, Beyotime Biotechnology). The cells were fixed in 4% paraformaldehyde at room temperature for 2 min. After washing three times with PBS, the cells were incubated in AP staining reagent at room temperature for 30 min in the dark. After washing three times with PBS, images were taken with a Leica DMI8 microscope.

### Immunofluorescence staining

The cells were fixed in 4% paraformaldehyde for 30 min and then washed three times with PBS. The cells were then incubated with perforation buffer containing 5% BSA and 0.2% Triton X-100 for 3 h, and the primary antibody against Sox2 (66411-1-Ig, Proteintech, 1:500) was added and incubated overnight at 4 °C. The next day, after being washed three times with PBS, the cells were incubated with a secondary antibody and Hoechst 33342 (Invitrogen, H3570,1:10,000) for 1 h at 37 °C. The cells were photographed under a Leica DMI8 microscope. The fluorescence intensity was analyzed with ImageJ according to the User Guide.

### Western blot

Cells were lysed in ice-cold RIPA cell buffer (P0013B, Beyotime Biotechnology) supplemented with PMSF (ST507, Beyotime Biotechnology). The protein samples were then separated *via* SDS‒PAGE (C671102, Sangon Biotech) and electrotransferred to a PVDF membrane (IPVH00010, Merck Millipore). Subsequently, the PVDF membrane was blocked with 5% skim milk for 4 h. After blocking, the membrane was incubated with specific primary antibodies overnight at 4 °C and followed by incubation with an HRP-conjugated secondary antibody at room temperature for 1 h. Images were acquired with a Tanon-5200Multi chemiluminescence gel imaging system (Tanon). The primary antibodies used in this study are FLAG (SG110–26, GNI 1:1000), Prdm14(D121722, BBI, 1:1000), Dnmt1(381634, ZENBIO, 1:1000), Dnmt3a(R26690, ZENBIO, 1:1000), Dnmt3b(UPA62506, Gene Universal, 1:1000), Dnmt3l (UPA05797, Gene Universal, 1:1000), PKCα(R25382, ZENBIO, 1:1000), PKCβ(R25383, ZENBIO, 1:1000), PKCδ(R25384, ZENBIO, 1:1000), β-tubulin (200608, ZENBIO, 1:2000), Suv39h1(10574-1-AP, Proteintech, 1:1000), Suv39h2(11338-1-AP, Proteintech, 1:1000), H3K9me2(39239, Active Motif, 1:1000), H3K9me3(39161, Active Motif, 1:1000), H3K27me3 (39157, Active Motif, 1:1000), Ezh2(21800-1-AP, Proteintech, 1:1000), and Ezh1(20852-1-AP, Proteintech, 1:1000).

### Quantitative real-time PCR

Total RNA was extracted using the MolPure Cell Total RNA Kit (19221ES50, Yeasen). cDNA was synthesized from 1 μg of total RNA using Hifair III first Strand cDNA Synthesis SuperMix for qPCR (11141ES60, Yeasen) according to the manufacturer’s instructions. qRT‒PCR was carried out with ChamQ SYBR qPCR Master Mix (Q311–02, Vazyme) using a PikoReal Real-Time PCR machine (Thermo Fisher Scientific). The relative expression level was determined by the 2-ΔCq method and normalized to the Rpl19 expression. The primers used are listed in Table S5.

### ChIP

ChIP experiments were performed using a ChIP assay kit (P2078, Beyotime Biotechnology) according to the manufacturer’s protocol. H3K9me2, H3K9me3, RNA Pol II, or RNA Pol II–S2P antibodies were used for immunoprecipitation, and IgG was used as a negative control. ChIP enrichment was determined by qRT‒PCR. The primer sequences and locations within the promoter regions of *Prdm14* are listed in Table S6.

### Plasmid transfection and virus production

For gene overexpression, 2 μg of PB and 2 μg of transposon plasmids were transduced into cells using the Hieff Trans Liposomal Transfection Reagent (40802ES03, Yeasen) according to the manufacturer’s instructions. For lentivirus production, 2 μg of pLKO.1, 0.75 μg of VSVG, and 1.25 μg of psPAX2 were transfected into 293FT cells together. After 48 h of transfection, the virus supernatant was collected and added to the cell culture medium to infect the cells. The transfectants were selected by adding 2 μg/ml puromycin.

### Dot blot

Genomic DNA (gDNA) was extracted from the cells using a cell gDNA extraction kit (DP304, TIANGEN). The gDNA was denatured in 0.1 M NaOH for 10 min at 95 °C. Two microliters of the serially diluted gDNA were spotted on a nitrocellulose membrane and kept at 70 °C for 30 min. Afterward, the membrane was blocked with 5% skim milk for 1 h and incubated overnight at 4 °C with primary antibodies. On the second day, the membrane was incubated with HRP-conjugated secondary antibodies for 1 h at room temperature. Images were captured with a Tanon-5200Multi chemiluminescence gel imaging system (Tanon). The primary antibodies used in this study were against 5mC (61255, Active Motif, 1:1000) and 5hmC (39770, Active Motif, 1:1000).

### TMT/iTRAQ-labeled quantitative proteomics

Approximately 1 × 10^8^ 46C mESCs were grown in 100 cm dishes and treated with DMSO or 5 μm Go6983. After 24 h, the cells were lysed with 1 ml of RIPA buffer supplemented with protein inhibitors and harvested by using a cell scraper. The eppendorf tubes containing the cell lysate were immediately frozen in liquid nitrogen and subsequently sent to PTM BIO., Inc on dry ice for TMT/iTRAQ-labeled quantification.

### Accession number

Our RNA sequencing data have been deposited in the GEO database under accession number GSE185220.

### Statistical analysis

The number of biological replicates is stated in each legend. All the data are reported as the mean±SD. The data were visualized with GraphPad Prism 8. Two paired Student’s *t* test or one-way ANOVA with Sidak’s multiple comparisons test was used to determine the significance of differences in the following comparisons. *p* < 0.05 indicated statistical significance.

## Data availability

The datasets used and/or analyzed during the current study are available from the corresponding author on reasonable request.

## Supporting information

This article contains supporting information.

## Conflict of interest

The authors declare that they have no conflict of interest with the contents of this article.
