# Supplementary material for: Inhibition of protein kinase C increases Prdm14 level to promote self-renewal of embryonic stem cells through reducing Suv39h-induced H3K9 methylation
Source: J Biol Chem. 2024 Feb 2;300(3):105714. doi: 10.1016/j.jbc.2024.105714 (PMC10909794; doi:10.1016/j.jbc.2024.105714)
Supplement: Supporting information [file mmc1.pdf]

Supporting Information

Figure S1

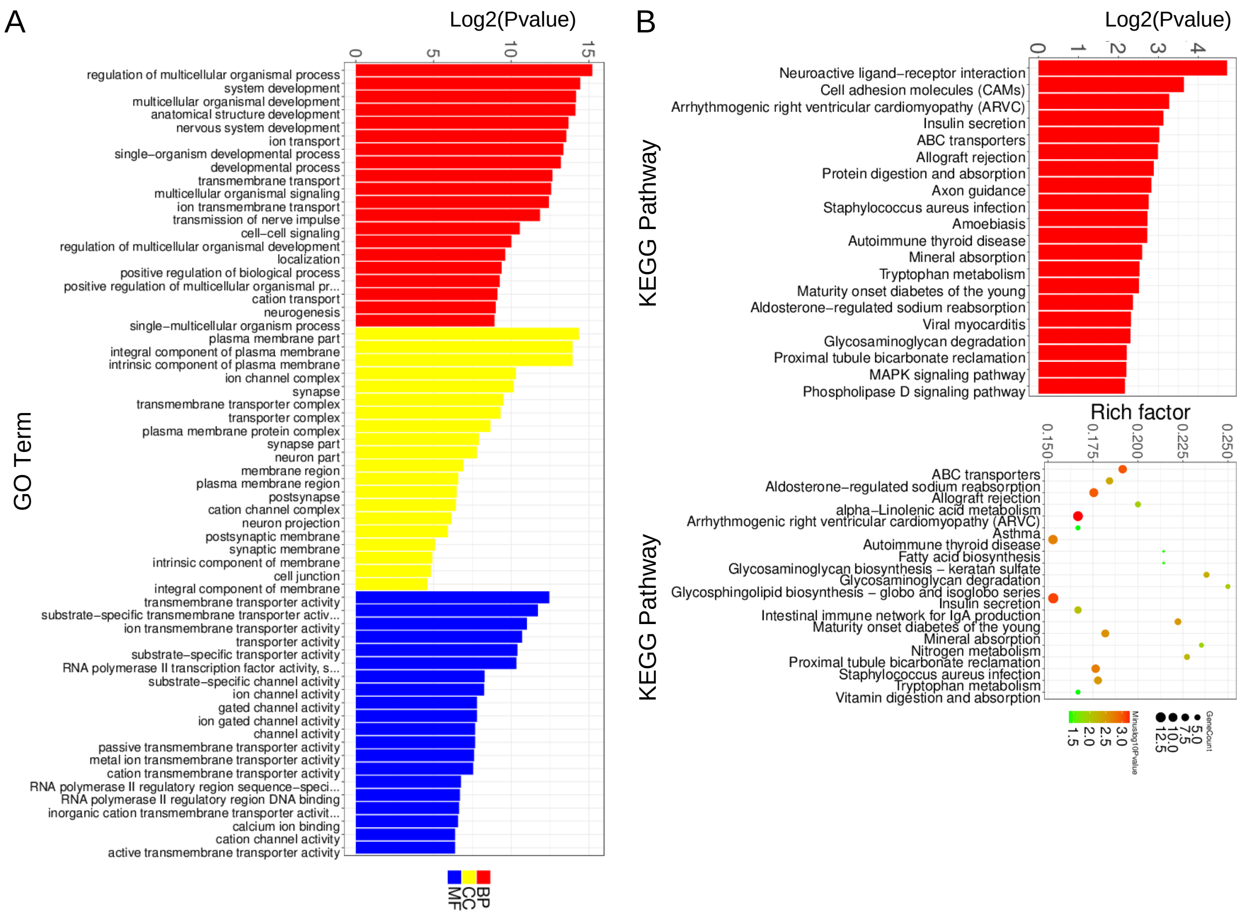

Figure S1. GO and KEGG analyses of the Go6983-mediated DEGs

(A) GO enrichment analysis of the differentially expressed genes mediated by Go6983.
(B) KEGG enrichment analysis of the differentially expressed genes mediated by Go6983.

Figure S2

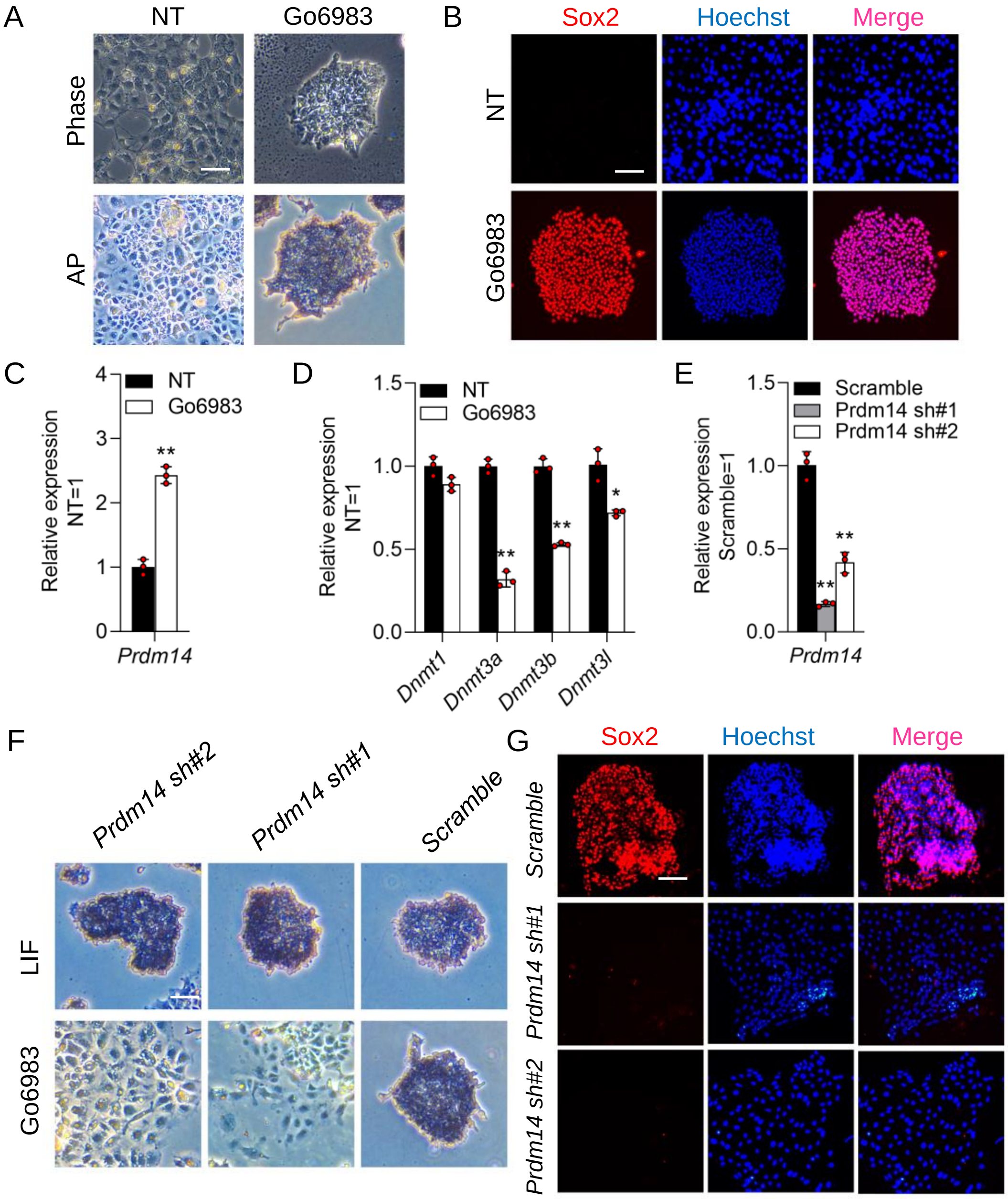

Figure S2. *Prdm14* mediates the function of Go6983 in human iPSCs

(A,B) Analysis of AP activity and Sox2 expression in hiPSCs cultured in N2B27/KSR-containing medium treated with or without Go6983 for 8 days. Scale bar, 100 μm.

(C,D) qRT–PCR analysis of the expression of *Prdm14*, *Dnmt1*, *Dnmt3a*, *Dnmt3b,* and *Dnmt3l* in hiPSCs treated with Go6983 for 24 h. Data represented as the mean ± SD (N = 3 biological replicates). *p < 0.05, **p < 0.01 versus NT, as determined by two paired Student's t test.

(E) qRT–PCR analysis of *Prdm14* in *scrambled* shRNA- and *Prdm14* shRNA hiPSCs. Data represented as the mean ± SD (N = 3 biological replicates). **p < 0.01 versus Scramble, as determined by one-way ANOVA with Sidak's multiple comparisons test.

(F,G) Analysis of AP activity and Sox2 expression in *scrambled* shRNA- and *Prdm14* shRNA-expressing hiPSCs treated with Go6983 for 8 days. Bar, 100 μm.

Figure S3

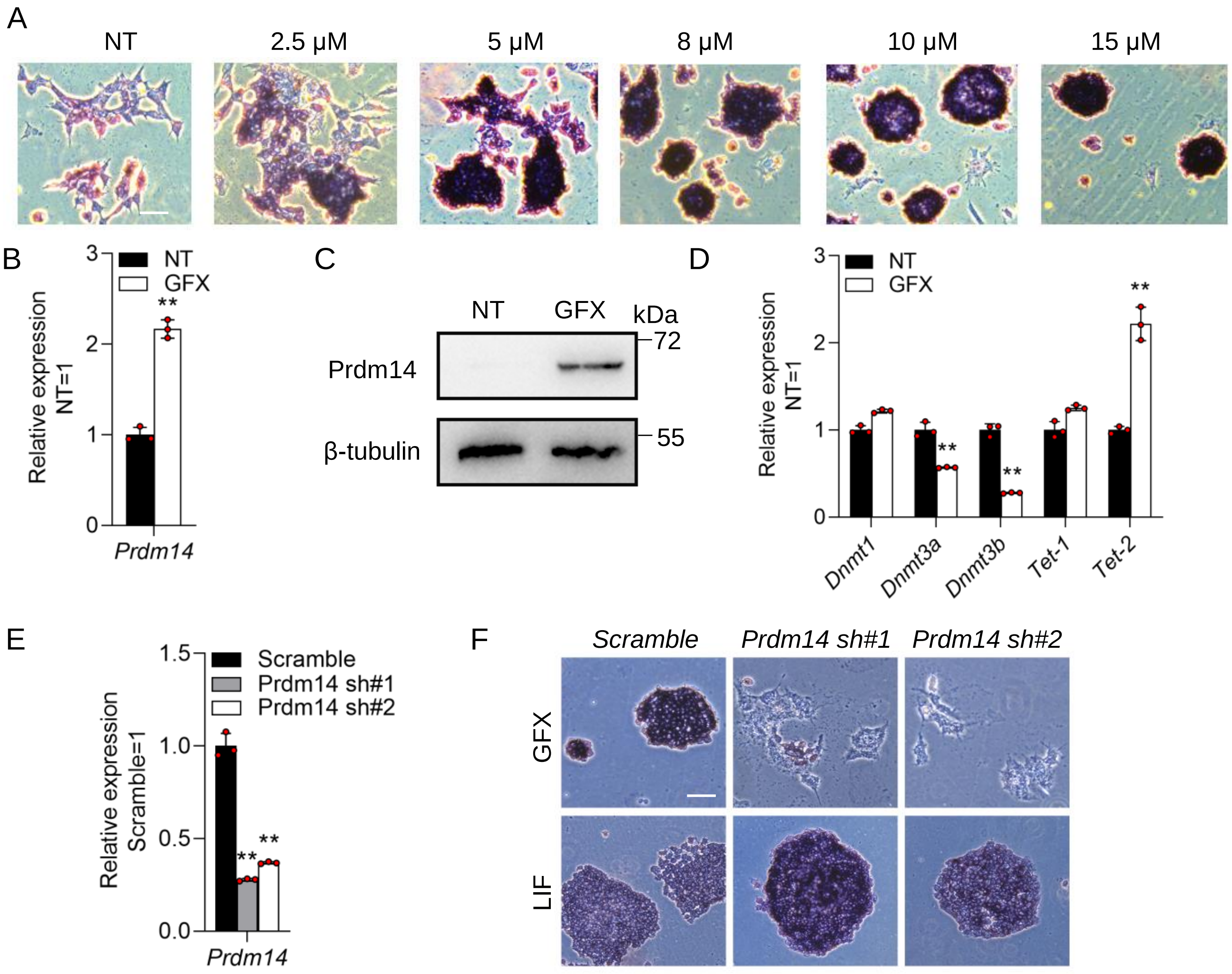

Figure S3. *Prdm14* mediates the self-renewal promoting effect of GFX in mESCs

(A) AP staining of mESCs treated with different concentrations of GFX for 8 days. Scale bar, 100 μm.

(B) qRT–PCR analysis of *Prdm14* expression in mESCs cultured in serum-containing medium in the presence or absence of GFX for 24 h. Data represented as the mean ± SD (N = 3 biological replicates). *p < 0.05, **p < 0.01 versus NT, as determined by two paired Student's t test.

(C) Western blot analysis of Prdm14 protein levels in 46C mESCs treated with or without GFX for 24 h.

(D) qRT–PCR analysis of *Dnmt1*, *Dnmt3a*, *Dnmt3b*, *Tet1,* and *Tet2* expression levels in mESCs in the presence or absence of GFX for 24 h. Data represented as the mean ± SD (N = 3 biological replicates). *p < 0.05, **p < 0.01 versus NT, as determined by two paired Student's t test.

(E) qRT–PCR analysis of *Prdm14* transcripts in 46C mESCs infected with *scrambled* shRNA or *Prdm14* shRNA lentivirus. Data represented as the mean ± SD (N = 3 biological replicates). *p < 0.05, **p < 0.01 versus Scramble.

(F) AP staining of *scrambled* RNA- and *Prdm14* shRNA-expressing cells cultured in serum-containing medium supplemented with GFX. Bar, 100 μm.

Figure S4

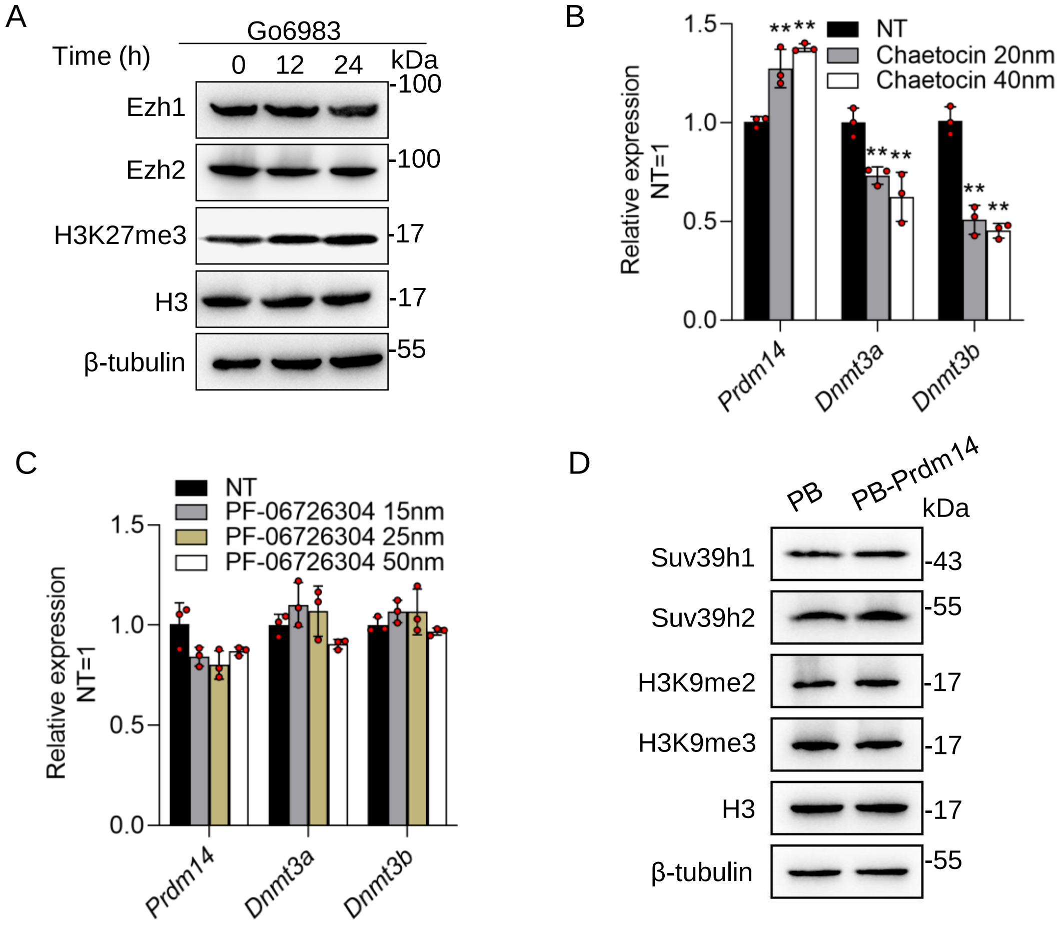

Figure S4. Effects of chaetocin and PF-06726304 on expression of the *Prdm14* and *Dnmt3* genes

(A) Western blot analysis of Ezh1, Ezh2, H3K27me3, and H3 protein levels in 46C mESCs treated with Go6983 for 12 or 24 h.

(B) qRT–PCR analysis of the expression of *Prdm14*, *Dnmt3a,* and *Dnmt3b* in 46C mESCs treated with 20 nm or 40 nm chaetocin for 24 h. Data represented as the mean ± SD (N = 3 biological replicates). $^{*}p < 0.05$, $^{**}p < 0.01$ versus NT, as determined by one-way ANOVA with Sidak's multiple comparisons test.

(C) qRT–PCR analysis of the expression of *Prdm14*, *Dnmt3a,* and *Dnmt3b* in 46C mESCs treated with 15 nm, 25 nm or 50 nm PF-06726304 for 24 h. Data represented as the mean ± SD (N = 3 biological replicates). $^{*}p < 0.05$, $^{**}p < 0.01$ versus NT, as determined by one-way ANOVA with Sidak's multiple comparisons test.

(D) Western blot analysis of the levels of Suv39h1, Suv39h2, H3K9me2 ,H3K9me3, and H3 in 46C mESCs overexpressing *empty vector PB* or *Flag-tagged Prdm14*.

Table S1. Proteins regulated by Go6983 and identified by LC-MS

Gene name	GO/DMSO Ratio	Regulated Type	Subcellular localization
Clk1	1.536	Up	nucleus
Hexa	1.502	Up	extracellular
Lrrc14	1.443	Up	mitochondria
Fdft1	1.421	Up	cytoplasm
Pros1	1.42	Up	extracellular
Med15	1.414	Up	nucleus
Zic2	1.388	Up	nucleus
Znf428	1.385	Up	nucleus
Pomp	1.36	Up	cytoplasm
Hmgcr	1.35	Up	plasma membrane
Cmc2	1.342	Up	extracellular
Prkcd	1.335	Up	cytoplasm
Clk4	1.332	Up	nucleus
Esco1	1.327	Up	nucleus
Slc27a1	1.321	Up	extracellular
Znf414	1.316	Up	nucleus
Snx13	1.314	Up	endoplasmic reticulum
Cd63	1.304	Up	plasma membrane
Emc4	1.303	Up	cytoplasm
Suv39h1	0.769	Down	nucleus
Vrtn	0.769	Down	nucleus
Rpl29	0.752	Down	nucleus
Pou3f1	0.75	Down	nucleus
Senp8	0.75	Down	nucleus
Slmap	0.736	Down	peroxisome
Sdf2l1	0.73	Down	extracellular
Xaf1	0.708	Down	extracellular

Table S2. List of primers for amplifying genes

Symbol	Forward sequence（5'-3'）	Reverse sequence（5'-3'）
	Mus musculus (house mouse)	
Prdm14	GAAGATCTATGGCCTTACCGCCCTCTGG	CCGCTCGAGCTAGCAGGTTTTATGAAGCCTCATG
PKCα	CGGCTTAAGATGGCTGACGTTTACCCGGCCAACGACTCC	CCGCTCGAGTCATACTGCACTTTGCAAGATTGGGTGCACAAACT
PKCβ	GGATGATCAATGGCTGACCCGGCTGCGG	CCGCTCGAGTTAGCTCTTGACTTCAGGTTTTAAA
PKCδ	GGATGATCAATGGCACCCTTCCTGCGCA	CCGCTCGAGTTAAATGTCCAGGAATTGCTCAAAC

Table S3. List of sequences used for gene knockdown

Symbol	ShRNA sequence（5'-3'）
Mouse Prdm14 sh#1	GCATACTCCGAACACACATCA
Mouse Prdm14 sh#2	ACCTTGAATTACAGGATTAAG
Human Prdm14 sh#1	CGTCCTATGGACACTACAGAA
Human Prdm14 sh#2	CGGATCCACATTCTTCATGTT
Mouse Suv39h1 sh#1	GCCTTTGTACTCAGGAAAGAA
Mouse Suv39h1 sh#2	CGAGTCCGTATTGAATGCAAA

Table S4. List of CRISPR/Cas9 sequences used for gene knockout

Symbol	Target sequence（5'-3'）
Mouse PKCα	AACCGCTTCGCCCGCAAAG
Mouse PKCβ	CAGTGCGTGTCGCCCGCAA
Mouse PKCδ	CTGGGCTCCCTGCAAGTTG

Table S5. List of primers used for qRT-PCR analysis

Symbol	Forward sequence （5'-3'）	Reverse order （5'-3'）
	Mus musculus (house mouse)	
RPL19	GACGGAAGGGCAGGCATATG	TGTGGATGTGCTCCATGAGG
Dnmt1	ATGGCGTCATAGCCCATAAG	CTGCACAGGAACAGACTCCA
Dnmt3a	ACCAGGCCACCTACAACAAG	GGTTCGCTTTCTCTTTCGGG
Dnmt3b	TGGTGATTGGTGGAAGCC	AATGGACGGTTGTCGCC
Dnmt3L	ATGGACAATCTGCTGCTGACTG	CGCATAGCATTCTGGTAGTCTCTG
Tet1	CCATTCTCACAAGGACATTCAC	GCAGGACGTGGAGTTGTTCA
Tet2	GCCATTCTCAGGAGTCACTGC	ACTTCTCGATTGTCTTCTCTATTGAG
Oct4	AGAGGGAACCTCCTCTGAGC	TTCATGTCCTGGGACTCCTC
Nanog	GTCTGATTCAGGGCTCAGCA	AAGGCTTCCAGATGCGTTCA
Tfcp2l1	AGGTGCTGACCTCCTGAAGA	CAGGCTGTTATCCCCACTGT
Prdm14	AAGCCTTTGCATCTCATGCT	AGGAAGCCTTTCCCACAAAT
Tbx3	CAGCTCACACTGCAGTCCAT	GAGACAGCAGGAGAGGATGC
Pou3f1	GTTCTCGCAGACCACCATCT	CTTCTCCAGTTGCAGGCTGT
Myc	CAACGTCTTGGAACGTCAGA	TCGTCTGCTTGAATGGACAG
Gbx2	ATTTGCCTGGTCAGACTGCT	GCCTTGACACGTTTCCACTT
Nr5a2	GCCCTGCTGGACTACACAAT	CCCGTTCAGGTGCTTGTAGT
PKCα	CTCGAGCAGGAAGTGGTAGG	TTGGGACCAGGGACTGTTAG
PKCβ	TCTGACCCCTTGTCCACTTC	GAAGTTGAGCCAGCATAGCC
PKCδ	GAAAAGACAGGCCGAGAGTG	TGAACCTGGGTAGGAAGTGG
PKCγ	CCCTGCACATTACACACCAC	CCCCTTCATACATCGGAGAA
PKCζ	TTGAATTGAACCCTGTTGTCCC	GAAACCCGTTCTCCCATCCACC
Suv39h1	CCTGCACAAGTTTGCCTACA	AGTGCGGAAGATGCAGAGAT
Ezh2	AGACGTCCAGCTCCTCTGAA	CATCCTCAGTGGGAACAGGT
	Homo sapiens (human)	
PRDM14	ACAGCCAAGCAATTTGCACTAC	TTACCTGGCATTTTCATTGCTC

Table S6. List of primers used for ChIP analysis

Symbol	Forward sequence（5'-3'）	Reverse sequence（5'-3'）
	Mus musculus (house mouse)	
-257~+1	GAGGGAGGGAGGAACAGAGGGA	GCACCCTCCCTCATTCTCCTTCA
-438~-238	GAAGGAGAATGAGGGAGGGTGC	CACACAGACCTTTGTTTTGTTTGCT
-628~-418	CAAACAAAACAAAGGTCTGTGTGGA	CGCCAAATACCCCCCTCCTTC
-809~-609	GGATGATCAATGGCACCCTTCCTGCGCA	CCGCTCGAGTTAAATGTCCAGGAATTGCTCAAAC
-989~-789	GAAGCACACACTGGTATCTGTGTAAC	GAAGGCAGCCTGTTAACTTCTACC
-1154~-978	GAAGTTAACAGGCTGCCTTCTTGG	TGTTTTGTCTGTGCACCAGTTGGT
-1365~-1164	ACTGGTGCACAGACAAAACAGCC	CCCCTCCCTTCTCCCTATTCTTTAC
-1510~-1354	AGAATAGGGAGAAGGGAGGGGAAG	GCAGCGCTTTATTTTCCGAAACGAA
-1721~-1521	TTTCGGAAAATAAAGCGCTGCCTCA	GAGGCGCATGAGAAGAGCAGGAATA
-1904~-1701	ATTCCTGCTCTTCTCATGCGCC	GAGTGAGGAGAGAGACTCAGTCTCTCT
-2087~-1885	ACTGAGTCTCTCTCCTCACTCATCA	ACTGTGCTCAACGACTGCATTTTTA
-2241~-2278	AATGCAGTCGTTGAGCACAGTTAC	GAACCAGGTAGGCTGAGAAGTTAGG
-2452~-2252	ACTTCTCAGCCTACCTGGTTCTCT	ACACACACAGACACACAGACACAC
-2632~-2432	TACTGCCTCCCTCTCCAAGTACC	GTAGCCTTCGGGGTAGGTCCC
-2810~-2618	TGTTTAGTTCTCACGCCGACACTTC	CTTGCACAGGCAGGTGTTGTT
-3001~-2789	CAACACCTGCCTGTGCAAGATG	TACTTGGAGAGGGAGGCAGTAGAAG
Prdm14_E1	CAGAACTCTCTGTGGGAACCA	GTCTCTGCCTCATGCAGACCTCATT
Prdm14_E2	TTCCCACATCCGAGAGTTGTCC	TTCCCACATCCGAGAGTTGTCC
Prdm14_E3	ATTTTTGAAGATGGTCACCTGA	CTTCAGAGGACTCAGACAGCTGCT
Prdm14_E4	AGTCTGCAGAAGGTTACAGAT	CAGGTAGGGCCGGTGCCTCTCATG
Prdm14_E5	TGTTCAACCTGTGGGAAAAGTT	CTTTGTACAGTACACGCACTGGTAT
Prdm14_E6	AAGTTCACTGCCTCCAGCATAC	CTAGCAGGTTTTATGAAGCCTCATG
Prdm14_E7	ACAAGGCGAACTACCTGCCCC	CGAAAAGCATCAAGAGGGGCCATC
